# Behavioral Drivers of Digital Participation: Security Trust, Outcome Efficacy, and Procedural Cues in South Korea

**DOI:** 10.3390/bs16060881

**Published:** 2026-06-01

**Authors:** Roksolana Kanzamanova, Seunghwan Myeong

**Affiliations:** Department of Public Administration, Inha University, Incheon 22212, Republic of Korea; escbit@icloud.com

**Keywords:** digital participation, behavioral intention, security trust, outcome efficacy, digital ability, survey experiment, South Korea

## Abstract

Digital participation depends not only on the formal availability of online engagement channels but also on how citizens interpret the safety, usefulness, and feasibility of participation. This article examines whether willingness to engage digitally is shaped more strongly by procedural platform cues or by underlying behavioral beliefs about security, efficacy, and personal capability. Using a survey of 500 adults in South Korea and a 2 × 2 survey-embedded vignette experiment, the study varies participation threshold (50 vs. 500 supporters) and response specificity (generic response vs. concrete action plan and timeline). The direct experimental effects are small and statistically non-significant, indicating no detectable moderate shift in stated willingness within this vignette design. In contrast, baseline participation intention, perceived outcome efficacy, and digital ability are consistently associated with scenario-based willingness to participate, while security trust is positively associated with baseline readiness to engage. The findings suggest that digital participation is better understood as a behavioral decision shaped by perceived risk, expected consequences, and self-assessed capability than as a simple response to procedural design alone.

## 1. Introduction

Despite the widespread availability of digital channels, citizen participation online remains limited. The mere provision of digital platforms does not guarantee engagement; rather, behavioral factors such as trust, perceived consequences, and personal ability often shape decisions to engage. As governments expand petition systems and participation portals, understanding these drivers becomes increasingly important. Engagement depends on individuals’ perceptions of risks and demands. Citizens may avoid digital participation if they distrust information security, question the platform’s impact, or lack confidence in using the system.

Engagement in digital systems depends on factors beyond simple access. Prior studies suggest that trust in digital systems, perceived utility, and confidence in personal digital abilities are often associated with digital participation, although these relationships may vary by demographic and technical-skill differences (Ajzen, 1991; Carter & Bélanger, 2005; Li & Shang, 2023; Porumbescu, 2016a, 2016b; Tolbert & Mossberger, 2006; Venkatesh et al., 2003; Welch et al., 2005). In voluntary civic contexts, perceptions of risk, futility, or difficulty frequently hinder engagement. Digital participation demonstrates how cues, risk perceptions, efficacy beliefs, and self-confidence interact to shape willingness to act.

South Korea serves as a rigorous test case for analyzing barriers to digital participation. As a high-capacity digital government with advanced infrastructure and widely recognized official platforms, South Korea provides an environment in which procedural cues can be critically evaluated (Anti-Corruption and Civil Rights Commission, n.d.; OECD, 2025; Office of the President of the Republic of Korea, 2022; Sung & Lee, 2024; UN DESA, 2024). This context clarifies the significance of procedural factors in optimal settings.

Digital participation systems are commonly structured by two procedural cues: the supporter threshold required for an idea’s official review and the specificity of official responses. These cues provide citizens with visible signals regarding attainability and institutional follow-through. A low threshold indicates a feasible review, while a concrete response signals recognizable action.

Citizens’ willingness to participate may be constrained if participation does not appear safe, effective, or accessible. Concerns regarding data protection, ambiguous effectiveness, and low digital confidence can deter engagement, even when processes are transparent or responses are promised (Carter & Bélanger, 2005; Naranjo-Zolotov et al., 2019). Effective participation requires not only procedural reform but also trust, efficacy, and capability.

Empirical evidence from a sample of 500 Korean participants reveals limited direct effects of procedural cues on willingness to participate. Lowering thresholds or promising concrete responses did not result in significant increases in engagement. Instead, baseline intention, outcome efficacy, digital ability, and security trust predict willingness, with these variables interpreted associatively. The novelty of the article lies in testing procedural platform cues together with behavioral belief and resource variables in a high-capacity digital-government setting, thereby clarifying why participation may remain limited even when formal online channels are available.

This article advances three main arguments. First, it frames digital participation as an issue of behavioral intention rather than solely a design question. Second, it demonstrates that procedural openness is distinct from the belief that participation matters. Third, it contends that trust in security and digital ability are fundamental to participation. Willingness to engage depends on more than procedural rules and official responses.

The following sections develop a behavioral framework for digital participation. The article connects procedural cues to attainability, security trust to risk, efficacy to outcomes, and digital ability to action. Subsequent sections detail the research design, present results, and discuss implications for behavioral engagement under conditions of uncertainty.

## 2. Theoretical Background and Hypotheses

### 2.1. Procedural Cues as Behavioral Signals

Digital participation increases consultation opportunities and lowers engagement costs (Shin et al., 2024; Weigl et al., 2024). From a behavioral perspective, these platforms serve as environments that communicate institutional intent. Because institutional commitment is not directly observable, citizens infer the value of participation from observable cues such as review thresholds, response rules, and the seriousness of administrative follow-through (Macintosh, 2004; Sæbø et al., 2008; Simonofski et al., 2021). Behavioral intention is shaped by both formal opportunities and interpretations regarding the attainability and meaningfulness of actions (Ajzen, 1991; Sheeran, 2002).

Thresholds and the specificity of responses are significant when they influence perceptions of the attainability and usefulness of participation. For example, lower thresholds reduce the collective-action burden and may signal that review is achievable. Concrete responses clarify institutional actions, timelines, and procedures for handling proposals. Research on e-participation and transparency demonstrates that such cues can facilitate engagement, although their effects depend on context and citizen interpretation (Kim & Lee, 2012; Mertes, 2022; Zhao et al., 2023).

**Hypothesis 1.** 
*A lower participation threshold increases citizens’ willingness to participate through a digital platform.*

**Hypothesis 2.**  *A concrete administrative response increases citizens’ willingness to participate through a digital platform.*

**Hypothesis 3.**  *A concrete administrative response offsets the negative effect of a high participation threshold.*

### 2.2. Security Trust as Perceived Risk

Procedural access is not enough if participation feels unsafe. Digital participation often requires login credentials, identity verification, contact information, or other forms of data disclosure. Accordingly, security trust can be understood as a perceived-risk condition for action. Citizens may avoid even convenient platforms if they fear unauthorized data sharing, weak privacy protection, or unclear accountability for digital harms (Carter & Bélanger, 2005; Kleizen et al., 2023; Saldanha et al., 2022). This is behaviorally important because civic participation is voluntary: when perceived risk rises, citizens can withhold engagement entirely.

Security trust is therefore expected to matter especially for baseline participation intention. Before people evaluate the details of a particular proposal opportunity, they must first believe that digital participation is a sufficiently safe activity to consider. Research on technology acceptance and e-government uptake similarly suggests that trust in the digital environment is central to whether citizens are willing to enter a system at all (Carter & Bélanger, 2005; Tolbert & Mossberger, 2006).

**Hypothesis 4.**  *Security trust is positively associated with baseline digital participation intention.*

### 2.3. Outcome Efficacy and Expected Consequences

A second belief is outcome efficacy, defined as the perception that participation leads to visible changes, concrete explanations, or administrative action. Outcome efficacy is analytically distinct from procedural responsiveness. A platform may be procedurally open by providing accessible rules and timely replies while still failing to persuade citizens that participation will have meaningful consequences. This distinction is central from a behavioral standpoint because willingness depends not only on whether a process exists, but also on whether action seems worth the time and effort required to participate (Ajzen, 1991; Alarabiat et al., 2021).

Outcome efficacy should be especially important for scenario-based willingness. The vignette asks whether respondents would propose or sign an idea about public transportation. Such willingness requires more than approval of digital government in the abstract. It requires the expectation that participation can matter. Visible results, explanations for rejection, and evidence that prior input influenced decisions should therefore be associated with higher willingness to engage (Karkin & Cezar, 2024; Luna et al., 2024; Mertes, 2022).

**Hypothesis 5.**  *Outcome efficacy is positively associated with scenario-based participation willingness, net of procedural design.*

### 2.4. Digital Ability and Baseline Intention

**Figure 1:**
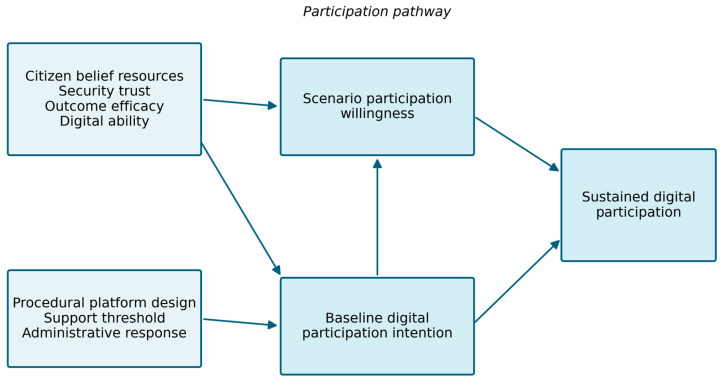
Conceptual Model. Note. The design factors are experimentally manipulated. Belief-resource pathways are estimated using observed scales and, in the robustness analysis, latent variables.

Digital ability and baseline participation intention determine whether platform opportunities are effectively translated into action. Although a system may appear accessible and potentially beneficial, citizens require sufficient confidence to navigate the platform, comprehend its requirements, and complete participation tasks. This perspective aligns with broader research on behavioral intention and technology adoption, which identifies self-assessed capability and prior readiness as significant predictors of voluntary engagement (Sheeran, 2002; Venkatesh et al., 2003). Recent research on mobile-banking adoption similarly emphasizes social influence, compatibility, digital self-efficacy, and perceived cost as factors that shape adoption of digital services, thereby underscoring the importance of capability and perceived barriers in platform use (Addula, 2025). In digital participation contexts, these resource-related beliefs may diminish the perceived importance of procedural features, as citizens who feel capable and predisposed to engage are more likely to participate across scenarios. Figure 1 summarizes these hypothesized pathways.

**Hypothesis 6.**  *Digital ability and baseline participation intention are positively associated with scenario-based participation willingness and reduce the apparent importance of procedural design features.*

## 3. Materials and Methods

### 3.1. Sample, Design, and Reporting Transparency

This article analyzes a de-identified secondary survey dataset of 500 adults in South Korea. The available questionnaire documentation identifies the Korea Local Administration Institute as the organizing institution and Research Lab Co., Ltd. as the survey agency. The survey instrument is dated November 2025 and indicates an expected response time of approximately 12–15 min. The questionnaire document preserves exact Korean wording for the scale items and the vignette frame; Appendix A reports the item-code mapping, exact Korean item wording, scale composition, and four condition-specific vignette versions used for transparent reporting. The questionnaire gathered information on demographics, digital skills, political views, perceptions of transparency, trust, integrity, administrative responsiveness, security trust, baseline digital participation intention, past participation, outcome efficacy, and responses to randomized vignettes. The analytic sample is balanced by gender and region, with each of the four experimental groups comprising 125 respondents. Analyses are unweighted and are interpreted as evidence from a balanced adult survey sample, rather than a nationally representative probability sample. The available materials state that responses were anonymous and used only for research/statistical purposes and that confidentiality was protected under Article 33 of the Korean Statistics Act. However, the available documentation does not report exact start and end field dates, the recruitment panel or sampling frame, response or completion rate, respondent compensation, a formal IRB approval or exemption number, full consent-form wording, or detailed attention-check and exclusion procedures. Consequently, the generalizability of the sample is considered a limitation, and empirical claims are restricted to randomized comparisons within the sample and associations among measured behavioral constructs.

### 3.2. Vignette Experiment

The study employed a 2 × 2 vignette experiment, which is effective for isolating the influence of specific institutional cues on behavioral judgments (Campbell, 2023). Campbell (2023) is cited as a methodological precedent for using vignette experiments in public-participation research; the data and public-transportation vignette analyzed here are from the present South Korean survey, not from Campbell’s dataset. Participants were instructed to envision proposing an idea to improve public transportation. The scenario randomly manipulated two aspects of the participation process: the number of supporters required for review (50 versus 500) and the nature of administrative follow-up (a generic response compared to a concrete action plan and timeline). For transparency, Appendix A and the revised survey supplement list the four assigned vignette versions separately: 50/generic, 50/concrete, 500/generic, and 500/concrete. Each of the four experimental conditions included 125 participants.

The threshold values serve as stylized representations of low- and high-burden cues, establishing a distinct tenfold difference in the number of supporters required for official review. This approach avoids suggesting that any single value characterizes all Korean digital participation systems. Similarly, the concrete-response manipulation isolates response specificity without replicating an entire administrative workflow. While these design choices enhance internal validity, they restrict the generalizability of findings to real-world platform behavior.

### 3.3. Design Sensitivity and Manipulation Salience

The dependent variable in this experimental analysis is willingness to participate in the scenario, measured on a scale from 1 (very low) to 7 (very high). The primary treatment variables include high threshold (coded as 1 for 500 supporters and 0 for 50 supporters), concrete response (coded as 1 for a concrete action plan and timeline and 0 for a generic response), and their interaction. As no separate manipulation check was documented, non-significant treatment estimates are interpreted as an absence of evidence for direct effects within this vignette design, rather than as definitive evidence that participants noticed, understood, and disregarded the threshold and response cues.

Reporting design sensitivity establishes realistic boundaries for interpreting null findings. With a total sample size of *N* = 500 and 125 respondents per experimental cell, the design is sufficiently powered to detect moderate treatment effects, but less so for very small effects. Based on the observed scale dispersion from the treatment-only model, the approximate minimum detectable effect with 80% power is 0.48 points on the 1–7 willingness scale for main-effect contrasts and 0.68 points for simple two-cell contrasts. Consequently, the experiment provides meaningful evidence against moderate direct effects in this vignette, although it cannot exclude the possibility of small or context-dependent effects.

### 3.4. Measurement and Discriminant Validity

Scales were calculated by averaging the relevant survey item responses for each construct. Transparency amplitude was measured using TA1 through TA4, trust coherence with TCpre1 through TCpre4, integrity perception with CPI1 through CPI3, administrative responsiveness with R1 through R3, and security trust with SECpre1 through SECpre5. Baseline participation intention was assessed using CP1 and CP3, both measured prior to the vignette. CP2 was excluded because it captures past participation behavior and was entered separately as a control variable. Scenario willingness was measured after the vignette and referred to a specific public transportation proposal or signing scenario. The ordering of measures, policy-domain specificity, and use of a distinct vignette outcome differentiate baseline readiness from scenario willingness, although common-method overlap cannot be fully eliminated within a single survey. Appendix A provides the item-code mapping, exact Korean item wording, scale composition, four vignette versions, and measurement limitations.

### 3.5. Estimation Strategy

The primary models employ OLS with HC3 heteroskedasticity-robust standard errors, as the 7-point vignette outcome facilitates interpretation as marginal differences in scale points. In addition to this approach, the analysis incorporates ordered-logit robustness checks, outcome-efficacy item robustness, variance inflation factor (VIF) diagnostics, a correlation matrix, and a latent structural equation modeling (SEM) robustness model. The SEM evaluates the measurement structure of latent constructs and the structural pathways from institutional beliefs and citizen resources to participation intention, outcome efficacy, and scenario participation willingness, in accordance with established guidance on latent-variable modeling and measurement validity (Bollen, 1989; Fornell & Larcker, 1981; Kline, 2016). These robustness checks address concerns related to measurement and functional form, but do not transform the observational predictors into randomized causal effects.

## 4. Results

Table 1 reports the sample profile: 50.2% male, 49.8% female, and exactly half reside in the capital region. The mean age is 48.64 years. The education profile is high, with 76.8% reporting college or higher education; population generalization is therefore made cautiously. In addition, 26.0% report having participated in a digital-government proposal, comment, or petition in the past year, indicating a substantial baseline of civic digital engagement within the sample.

Table 2 presents scale reliability (Cronbach, 1951; McDonald, 1999). Most scales have strong internal consistency: security trust (α = 0.926), integrity perception (α = 0.886), administrative responsiveness (α = 0.859), and trust coherence (α = 0.853). Participation intention is also reliable (α = 0.820). Outcome efficacy is the only borderline scale (α = 0.680; ω = 0.708), so it is interpreted with caution. The scale is retained because omega exceeds 0.70, deletion tests do not materially improve reliability, alternative item specifications reproduce the substantive association, and the SEMs outcome efficacy as a latent construct. Because the questionnaire document provides exact Korean item wording, Appendix A reports the wording used for the main scales and the vignette; the discriminant-validity assessment still rests on measurement timing, construct role, and robustness diagnostics because the same survey instrument collected several related perceptions.

Because outcome efficacy is both substantively central and measurement-vulnerable, the latent SEM is introduced early in the results narrative. Rather than treating alpha as the only evidence of measurement quality, the SEM estimates the outcome-efficacy factor from its indicators and tests whether that factor is associated with scenario willingness after accounting for participation intention, security trust, digital ability, past participation, and the experimental treatments. As shown below, the substantive conclusion is unchanged: outcome efficacy remains significant, whereas the procedural manipulations do not.

**Figure 2:**
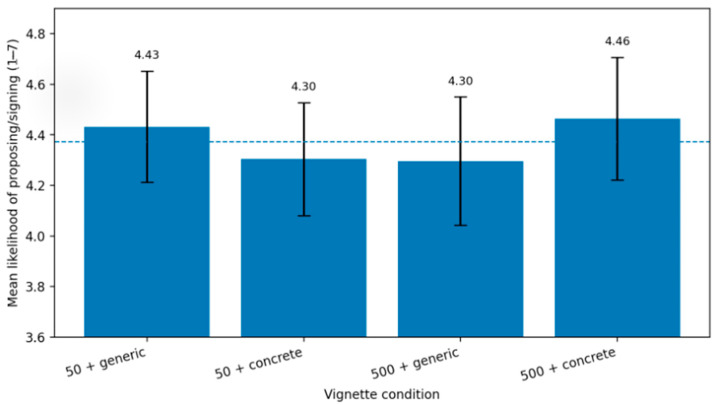
Participation Willingness by Vignette Condition. Note. Bars show condition means; error bars show 95% confidence intervals. The dashed line is the grand mean.

Figure 2 reports the mean likelihood of proposing or signing in each condition: 4.43 (50-supporter/generic-response), 4.30 (50-supporter/concrete-response), 4.30 (500-supporter/generic-response), and 4.46 (500-supporter/concrete-response). The similar means across all conditions indicate that the threshold and response-specificity cues did not produce detectable moderate direct shifts in behavioral willingness under this vignette. This pattern does not imply that platform context is irrelevant; rather, it indicates that procedural cues alone were not the strongest drivers of stated intention here.

Table 3 presents OLS models predicting scenario participation willingness. Model 1 includes only the experimental treatment variables, none of which are statistically significant. Model 2 adds demographic and resource controls; digital ability is positively associated with scenario willingness, while procedural treatments remain non-significant. Model 3 incorporates institutional belief and efficacy variables. The behavioral pattern is clear: baseline participation intention (b = 0.333, *p* < 0.001), outcome efficacy (b = 0.265, *p* = 0.001), and digital ability (b = 0.160, *p* = 0.001) are the strongest predictors. Because these variables are measured rather than randomized, the coefficients should be interpreted as associations, not as experimental proof of causal effects.

**Figure 3:**
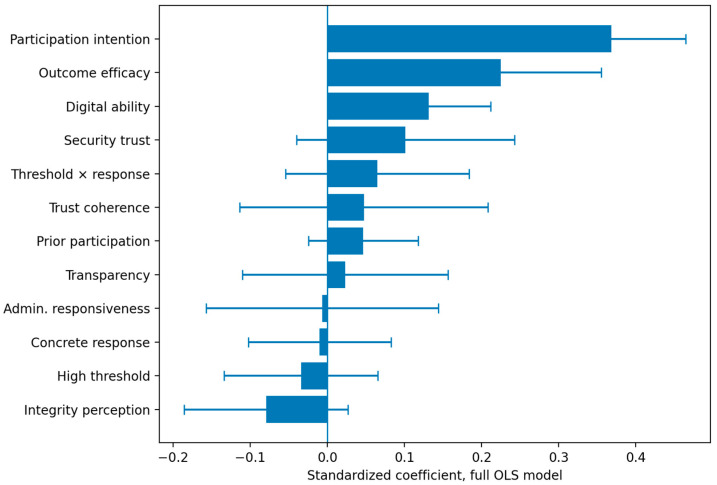
Standardized Coefficients in the Full Participation-Willingness Model. Note. Coefficients are from the fully standardized version of Model 3; error bars show 95% confidence intervals.

To further illustrate the pattern, Figure 3 reports standardized coefficients from the full OLS model. The standardized coefficient for baseline participation intention is the largest in the model (β = 0.368). Outcome efficacy (β = 0.225) and digital ability (β = 0.132) also show meaningful associations. In contrast, the direct experimental terms are small and imprecise. Taken together, these findings suggest that behavioral willingness in digital participation settings depends more on prior readiness, expected consequences, and perceived capability than on procedural cues alone.

Table 4 examines baseline participation intention. Security trust is positively associated with participation intention (b = 0.193, *p* = 0.021), supporting H4. Integrity perception is also significant (b = 0.159, *p* = 0.011), as are digital ability and past participation. Trust coherence and responsiveness are not significant after the highly correlated institutional belief variables are entered together. This pattern suggests that security trust functions primarily as an upstream condition of readiness: citizens who do not trust the digital environment may never reach the point at which platform-level cues become behaviorally salient.

Table 5 summarizes the hypothesis-level interpretation. The experimental hypotheses (H1–H3) are not supported because the threshold, response, and interaction terms are not statistically significant. The measured-association hypotheses are supported: security trust is positively associated with baseline participation intention (H4), outcome efficacy is positively associated with scenario willingness (H5), and digital ability and baseline participation intention are positively associated with scenario willingness (H6). These conclusions distinguish randomized treatment evidence from observed associations.

### 4.1. Correlations and Multicollinearity Diagnostics

Due to the conceptual proximity of the institutional belief variables, multicollinearity may attenuate individual coefficients in models that include transparency, trust coherence, integrity, administrative responsiveness, and security trust simultaneously. Correlations among these constructs are substantial, with the highest observed between trust coherence and administrative responsiveness (r = 0.76), administrative responsiveness and security trust (r = 0.76), and transparency and trust coherence (r = 0.76). Nevertheless, all variance inflation factors (VIFs) remain below the standard threshold of 5, with the highest being 3.92 for administrative responsiveness. This indicates that multicollinearity is moderate and does not pose a critical concern. Appendix B provides a summary of these diagnostics.

### 4.2. Latent SEM and Measurement-Error Robustness

Given that several predictors represent latent institutional beliefs, a structural equation model (SEM) is estimated to assess robustness and address measurement error. The SEM includes seven latent constructs: transparency amplitude, trust coherence, integrity perception, administrative responsiveness, security trust, participation intention, and outcome efficacy. Participation intention and outcome efficacy are regressed on the institutional belief variables, digital ability, and prior participation. Scenario willingness is regressed on the experimental factors, participation intention, outcome efficacy, security trust, digital ability, and prior participation. This modeling approach is particularly important for outcome efficacy, as SEM estimates its association with participation while accounting for measurement imperfections, rather than relying solely on the marginal reliability of a brief three-item scale.

The model fits the data well by conventional approximate-fit criteria: Comparative Fit Index (CFI) = 0.954, Tucker–Lewis Index (TLI) = 0.947, Root Mean Square Error of Approximation (RMSEA) = 0.047, and Standardized Root Mean Square Residual (SRMR) = 0.066. The chi-square test is significant, as expected, given the sample size and the many measured indicators. The key path pattern reproduces the OLS interpretation. Latent participation intention and latent outcome efficacy are significantly associated with scenario willingness, whereas the experimental treatment terms remain non-significant. Appendix B reports the available fit statistics and summarizes the SEM, ordinal-outcome, and outcome-efficacy robustness checks.

### 4.3. Ordinal and Outcome-Efficacy Robustness Checks

Appendix B addresses the ordinal nature of the 7-point dependent variable using ordered-logit robustness. The substantive conclusion is unchanged: procedural treatments remain non-significant, while baseline participation intention, outcome efficacy, and digital ability remain significant predictors. Appendix B shows that outcome efficacy is also robust to item deletions and single-item specifications, reducing the risk that the main association is an artifact of borderline alpha.

## 5. Discussion

### 5.1. Main Findings and Implications

The findings indicate a specific behavioral insight: procedural responsiveness alone was insufficient in this vignette. Although it is reasonable to anticipate increased citizen participation when thresholds are low and the government commits to a concrete response, these visible cues did not yield statistically significant moderate direct effects on stated willingness. The most robust correlates were baseline participation readiness, perceived outcome efficacy, and digital capability. From a behavioral science perspective, these results suggest that willingness to engage is influenced more by citizens’ evaluations of risk, consequence, and personal capability than by procedural cues alone.

However, this does not imply that thresholds or administrative feedback lack practical relevance. The experiment employed a concise vignette, did not include a separate manipulation check, and cannot exclude the possibility of small effects. A more defensible and behaviorally informative interpretation is that, within this design, procedural reforms alone produced no detectable moderate direct shift in stated willingness. Even if a platform reduces thresholds, participation may remain limited if citizens do not perceive the process as safe or consequential. This interpretation is consistent with previous research indicating that while convenience and simplicity can influence e-participation, perceived data protection and expected impact are central determinants of citizens’ willingness to engage (Mertes, 2022; Naranjo-Zolotov et al., 2019).

The intention-behavior gap further emphasizes this implication. Stated willingness in a scenario represents a lower threshold than actual participation, as respondents are not required to authenticate, disclose information, draft proposals, persuade others, or revisit the platform. Because procedural treatments do not significantly increase even low-cost stated willingness, their impact on actual participation is likely limited unless the platform also fosters security confidence and credibility regarding consequences. Therefore, platform designers should not assume that lowering thresholds or providing more specific responses will automatically result in increased participation without clear evidence that participation is both safe and meaningful (Ajzen, 1991; Sheeran, 2002).

This study also advances behavioral research on digitally mediated civic action. While much of the digital government literature focuses on adoption, satisfaction, or trust, the present findings indicate that willingness to participate is more accurately conceptualized as a belief-based decision-making process. Citizens appear to consider at least three questions before engaging: Is it safe? Will it matter? Can I do it? These questions correspond to security trust, outcome efficacy, and digital ability, respectively. The article thus links institutional platform design to broader behavioral constructs such as perceived risk, anticipated consequences, and self-efficacy in voluntary action contexts.

The results further elucidate the role of security trust. Security trust is significantly associated with baseline participation intention, indicating that it constitutes a component of the broader belief framework that enables digital participation. However, its direct effect in the full scenario model is not consistently significant, as intention and outcome efficacy explain much of the relationship. This pattern supports the view that security trust primarily functions upstream by influencing whether citizens are willing to enter the participatory environment.

For researchers and practitioners, these findings have practical implications. Digital participation platforms should not depend solely on lower thresholds or standardized response commitments. Instead, they should establish visible consequence pathways that demonstrate what has changed, explain why ideas were rejected, and clarify how personal information is protected. Participation systems should also minimize ability-related barriers by providing clear instructions, illustrative examples, and user-friendly interface design, as behavioral willingness depends on both institutional signals and users’ confidence in their ability to participate effectively.

### 5.2. Limitations and Future Research

Several limitations inform the interpretation of these findings and suggest avenues for future research. First, the dependent variable measures stated willingness rather than observed platform behavior. This distinction is important because actual participation involves multiple steps, including noticing the opportunity, logging in, trusting the authentication process, understanding the interface, allocating time, and perceiving that participation is worth any privacy or social risk. The well-documented intention-behavior gap likely results in smaller real-world effects compared to vignette-based effects (Ajzen, 1991; Sheeran, 2002). Given that procedural treatments do not substantially increase even stated willingness, their impact on observed participation may be even weaker. These findings support a cautious interpretation of the core claim: sustained participation likely depends on trust, efficacy, and capability, in addition to lower participation thresholds or more specific response incentives.

Second, the sample warrants cautious interpretation. While gender and regional distributions are balanced, the sample is highly educated, and the data documentation lacks information on field dates, recruitment platform, response and completion rates, compensation, consent wording, and ethical review. As a result, the unweighted analyses are best suited for internal comparisons within the survey sample rather than for generating nationally representative estimates. Future research should employ fully documented probability sampling, benchmark quotas, or post-stratification weights to enable valid population-level inferences.

Third, the outcome efficacy measure demonstrates borderline internal consistency. To address this, the manuscript reports omega in the main text and applies item-level robustness checks, alternative item specifications, and latent structural equation modeling to reduce reliance on a single composite score. Although Appendix A now reports the exact Korean wording of the outcome-efficacy items, the brief three-item format still limits how finely the construct can be separated into past impact, expected impact, feedback quality, attribution of change, and motivation for re-participation. Future studies should expand the outcome efficacy construct by including additional items that assess these dimensions separately.

Fourth, the vignette is limited to a single policy domain—public transportation—and a single participation format, namely proposing or signing an idea. Other domains, such as welfare, taxation, local development, environmental policy, or public safety, may yield different treatment effects. Additionally, the absence of a separate manipulation check in the study documentation means that null treatment estimates could result from weak salience, limited treatment realism, insufficient statistical power for small effects, or a genuine lack of direct treatment effects.

Fifth, the institutional belief variables exhibit high intercorrelations. Although correlation and variance inflation factor diagnostics suggest that multicollinearity is not prohibitive, future research should employ larger samples and more nuanced measurement models to better distinguish among transparency, responsiveness, integrity, trust coherence, and security trust.

Finally, while South Korea represents a theoretically valuable case, it is not universally generalizable. Comparative research should therefore examine whether similar patterns emerge in countries with lower digital government capacity, lower institutional trust, or differing privacy regimes.

## 6. Conclusions

Digital participation is frequently conceptualized as a procedural issue, wherein reducing thresholds and promising feedback are expected to increase citizen engagement. However, the findings of this study indicate that the behavioral dynamics are more complex. In a balanced 2 × 2 vignette experiment, neither lower thresholds nor concrete-response promises produced statistically significant moderate direct effects on stated willingness to participate. Instead, willingness to participate in scenarios was most strongly associated with baseline participation intention, perceived outcome efficacy, and digital ability. Additionally, security trust was positively correlated with baseline readiness to engage.

The primary implication is that digital participation extends beyond procedural design considerations. It also encompasses issues of security confidence, consequence credibility, and citizen capability. Platforms that enhance rules and response formats without addressing these broader factors may appear more responsive but are unlikely to achieve substantive increases in participation. For behavioral research, this article demonstrates that digitally mediated civic engagement can be conceptualized as an intention-formation process influenced by perceived risk, anticipated consequences, and self-assessed capability. In practice, the findings suggest that governments aiming to strengthen digital participation must address factors beyond procedural improvements.

## Figures and Tables

**Table 1:** Sample Characteristics.

Characteristic	Value	Share/Dispersion
*N*	500	100.0%
Male	251	50.2%
Female	249	49.8%
Capital region	250	50.0%
Non-capital region	250	50.0%
Age 18–29	69	13.8%
Age 30–44	124	24.8%
Age 45–59	141	28.2%
Age 60+	166	33.2%
College or higher	384	76.8%
Past digital-government participation	130	26.0%
Digital ability	Mean = 4.93	SD = 1.10
Political ideology	Mean = 5.48	SD = 1.81

Note. Capital region includes Seoul, Incheon, and Gyeonggi. Digital ability is measured from 1 = very low to 7 = very high. Political ideology is measured from 0 = conservative to 10 = progressive. No survey weights are applied in the main analyses.

**Table 2:** Construct Measurement and Reliability.

Construct	Indicators	k	Alpha	Omega/CR	AVE	Loading Range	Decision
Transparency amplitude	TA1, TA2, TA3, TA4	4	0.794	0.801	0.511	0.482–0.857	Retain
Trust coherence	TCpre1, TCpre2, TCpre3, TCpre4	4	0.853	0.860	0.613	0.557–0.870	Retain
Integrity perception	CPI1, CPI2, CPI3	3	0.886	0.887	0.724	0.797–0.899	Retain
Administrative responsiveness	R1, R2, R3	3	0.859	0.860	0.672	0.802–0.848	Retain
Security trust	SECpre1, SECpre2, SECpre3, SECpre4, SECpre5	5	0.926	0.927	0.717	0.774–0.870	Retain
Participation intention	CP1, CP3	2	0.820	0.820			Retain
Outcome efficacy	OE1, OE2, OE3	3	0.680	0.708	0.465	0.481–0.905	Retain with robustness checks

Note. α = Cronbach’s alpha. ω/CR is McDonald’s omega from a one-factor congeneric model for scales with three or more items; for the two-item participation-intention scale, alpha is reported as the reliability estimate. Outcome efficacy is retained because deletion does not materially improve reliability, omega exceeds 0.70, the coefficient is robust to alternative item specifications, and the latent SEM models the construct while accounting for measurement error.

**Table 3:** OLS Models Predicting Scenario Participation Willingness.

Predictor	Model 1	Model 2	Model 3
High threshold (500 vs. 50)	−0.136 (0.172)	−0.045 (0.161)	−0.091 (0.136)
Concrete response	−0.128 (0.161)	−0.023 (0.147)	−0.027 (0.126)
High threshold × concrete response	0.296 (0.241)	0.190 (0.222)	0.200 (0.188)
Male		0.200 † (0.113)	0.105 (0.097)
Age 30–44		0.211 (0.169)	0.216 (0.148)
Age 45–59		0.334 * (0.157)	0.245 † (0.139)
Age 60+		0.096 (0.171)	0.157 (0.149)
Capital region		−0.093 (0.110)	−0.016 (0.095)
College or higher		0.167 (0.151)	0.109 (0.119)
Digital ability		0.327 *** (0.056)	0.160 ** (0.050)
Political ideology		0.100 ** (0.036)	−0.013 (0.030)
Past participation		0.665 *** (0.116)	0.142 (0.111)
Transparency			0.028 (0.084)
Trust coherence			0.058 (0.100)
Integrity perception			−0.073 (0.050)
Administrative responsiveness			−0.007 (0.084)
Security trust			0.100 (0.071)
Baseline participation intention			0.333 *** (0.045)
Outcome efficacy			0.265 *** (0.078)
Constant	4.432 *** (0.113)	1.665 *** (0.372)	0.401 (0.322)
R^2^	0.003	0.205	0.457
*N*	500	500	500

Note. Entries are unstandardized coefficients with HC3 robust standard errors in parentheses. Reference age group is 18–29. † *p* < 0.10, * *p* < 0.05, ** *p* < 0.01, *** *p* < 0.001.

**Table 4:** Antecedents of Baseline Digital Participation Intention.

Predictor	Participation Intention
Male	0.086 (0.112)
Age 30–44	0.082 (0.191)
Age 45–59	0.072 (0.182)
Age 60+	0.021 (0.187)
Capital region	−0.032 (0.113)
College or higher	0.036 (0.142)
Digital ability	0.168 ** (0.059)
Political ideology	0.044 (0.035)
Past participation	0.695 *** (0.123)
Transparency	0.162 (0.099)
Trust coherence	0.114 (0.107)
Integrity perception	0.159 * (0.062)
Administrative responsiveness	−0.045 (0.091)
Security trust	0.193 * (0.084)
Constant	0.250 (0.370)
R^2^	0.339
*N*	500

Note. Dependent variable is the average of CP1 and CP3. OLS with HC3 robust standard errors. Reference age group is 18–29. * *p* < 0.05, ** *p* < 0.01, *** *p* < 0.001.

**Table 5:** Hypothesis-Level Summary of Findings.

Hypothesis	Expected Relationship	Empirical Result	Interpretation
H1	Lower participation threshold increases scenario willingness.	Threshold treatment is not statistically significant.	Not supported.
H2	Concrete administrative response increases scenario willingness.	Concrete-response treatment is not statistically significant.	Not supported.
H3	Concrete response offsets the negative effect of a high threshold.	Threshold × response interaction is not statistically significant.	Not supported.
H4	Security trust is positively associated with baseline participation intention.	Security trust is positive and significant in the baseline-intention model.	Supported as an association.
H5	Outcome efficacy is positively associated with scenario willingness.	Outcome efficacy is positive and significant in the full willingness model.	Supported as an association.
H6	Digital ability and baseline intention are positively associated with scenario willingness.	Digital ability and baseline intention are positive and significant in the full willingness model.	Supported as associations.

Note. H1–H3 are based on randomized vignette treatment estimates. H4–H6 are associations among measured variables and should not be interpreted as randomized causal effects.

## Data Availability

The data presented in this study are available on request from the corresponding author.

## Appendix A

### Measurement Item Codes and Documentation Limits

This appendix reports the exact Korean questionnaire wording available in the study documentation, together with English translations for reader convenience, analytic coding decisions, and remaining documentation limits. The Korean wording should be treated as the authoritative questionnaire wording, while English construct labels and English item translations are analytic translations. The exact wording for the main scale items and the vignette frame is available; however, full consent-form wording, exact field dates, recruitment-panel details, response/completion rates, compensation information, and detailed attention-check/exclusion wording remain unavailable.

**Table A1:** Measurement Item Codes and Documentation Limits.

Measure or Field	Original Korean Wording	English Translation
**Transparency amplitude** (TA1–TA4)	TA1. 내 의견이 실제 정책결정에 반영될 수 있다. TA2. 행정기관은 참여 결과가 어떻게 반영되었는지를 공개한다. TA3. 제안이 접수된 이후 처리 과정을 추적할 수 있다. TA4. 시민참여는 형식적 절차에 그치지 않는다.	TA1. My opinion can be reflected in actual policy decisions. TA2. Administrative agencies disclose how the results of participation are reflected. TA3. I can track the processing procedure after a proposal has been submitted. TA4. Citizen participation does not remain merely a formal procedure. **Interpretive implication:** Four pre-vignette items capture perceived transparency and traceability of participation outcomes.
**Trust coherence** (TC1–TC4)	TC1. 행정기관은 약속한 참여 절차를 실제로 이행한다. TC2. 시민 의견에 대한 답변이 정해진 기간 내에 이루어진다. TC3. 규정된 절차와 실제 대응이 일치한다. TC4. 앞으로도 유사한 참여가 일관성 있게 처리될 것으로 믿는다.	TC1. Administrative agencies actually carry out the participation procedures they have promised. TC2. Responses to citizens’ opinions are provided within the specified period. TC3. The prescribed procedures and actual responses are consistent. TC4. I believe that similar participation will continue to be handled consistently in the future. **Interpretive implication:** These items capture consistency between promised and actual participation procedures.
**Integrity perception** (CPI1–CPI3)	CPI1. 공직사회에 부패가 드물다. CPI2. 규칙이 지위나 연고와 상관없이 공정하게 적용된다. CPI3. 신고나 제보는 실제 제재로 이어진다.	CPI1. Corruption is rare in the public sector. CPI2. Rules are applied fairly regardless of status or personal connections. CPI3. Reports or tips lead to actual sanctions. **Interpretive implication:** These items measure perceived administrative integrity and fair rule application.
**Administrative responsiveness** (R1–R3)	R1. 제안이나 민원을 제출하면 합리적인 시간 내에 답변을 받는다. R2. 답변은 형식적인 내용이 아니라 구체적인 조치 내용을 담고 있 다. R3. 참여 임계치(서명·투표 수 등)는 현실적이다.	R1. When I submit a proposal or complaint, I receive a response within a reasonable time. R2. Responses contain specific action details rather than merely formal content. R3. Participation thresholds, such as the number of signatures or votes, are realistic. **Interpretive implication:** These items measure perceived responsiveness and feasibility of participatory procedures.
**Security trust** (SEC1–SEC5)	SEC1. 정부 온라인 플랫폼은 개인정보를 안전하게 보호한다. SEC2. 내 정보가 제3자에게 무단으로 공유될 가능성은 거의 없다. SEC3. 참여 과정에서 개인정보 보호 관련 안내가 명확히 제공된다. SEC4. 시스템 해킹이나 정보유출 사고가 발생하더라도 정부는 신속하게 대응한다. SEC5. 플랫폼 보안이 충분히 신뢰할 만하다고 느낀다.	SEC1. Government online platforms safely protect personal information. SEC2. There is little possibility that my information will be shared with third parties without authorization. SEC3. Clear guidance on personal information protection is provided during the participation process. SEC4. Even if a system hacking or information-leak incident occurs, the government responds quickly. SEC5. I feel that platform security is sufficiently trustworthy. **Interpretive implication:** Five pre-vignette items capture perceived data protection, privacy guidance, unauthorized sharing risk, incident response, and overall platform-security confidence.
**Baseline digital participation intention and past participation** (CP1–CP3)	CP1. 앞으로 3개월 내에 전자정부 플랫폼에 참여할 의향이 있다. CP2. 지난 1년간 실제로 제안·댓글·청원에 참여한 경험이 있다. (예/아니오) CP3. 이번 달에 참여를 위해 15분 이상 시간을 투자할 의향이 있다.	CP1. I intend to participate on an e-government platform within the next three months. CP2. In the past year, I have actually participated by submitting a proposal, posting a comment, or participating in a petition. (Yes/No) CP3. I intend to spend at least 15 min participating this month. **Analytic use:** CP1 and CP3 form the baseline participation-intention composite. CP2 captures past participation and is entered separately as a control variable.
**Outcome efficacy** (OE1–OE3)	OE1. 나의 참여로 정책이 실제로 개선된 사례가 있다. OE2. 눈에 보이는 결과가 있을 때 재참여 의지가 높아진다. OE3. 제안이 채택되지 않더라도 이유를 알려준다.	OE1. There has been a case in which my participation actually improved policy. OE2. My willingness to participate again increases when there are visible results. OE3. Even when a proposal is not adopted, the reason is provided. **Interpretive implication:** The scale captures perceived policy improvement, motivation from visible outcomes, and explanatory feedback. Reliability is borderline, so the manuscript reports robustness checks and SEM measurement-error sensitivity.
**Scenario participation willingness and four assigned vignette versions**	**Question stem:** 가정: 귀하가 ‘대중교통 개선 아이디어’를 제안하려 합니다. 이 상황에서 실제로 제안하거나 서명할 가능성은 얼마나 됩니까? (1–7점 척도) **50/generic:** 제안 검토를 위해 필요한 동의 수는 50명이며, 시청은 일반적인 답변만 게시합니다. **50/concrete:** 제안 검토를 위해 필요한 동의 수는 50명이며, 시청은 구체적 실행계획과 일정을 공개합니다. **500/generic:** 제안 검토를 위해 필요한 동의 수는 500명이며, 시청은 일반적인 답변만 게시합니다. **500/concrete:** 제안 검토를 위해 필요한 동의 수는 500명이며, 시청은 구체적 실행계획과 일정을 공개합니다.	**Question stem:** Assume that you are going to propose an idea to improve public transportation. In this situation, how likely are you to actually make the proposal or sign/support it? (1–7 scale) **50/generic:** The number of supporters required for the proposal to be reviewed is 50, and the city government posts only a general response. **50/concrete:** The number of supporters required for the proposal to be reviewed is 50, and the city government discloses a concrete action plan and timeline. **500/generic:** The number of supporters required for the proposal to be reviewed is 500, and the city government posts only a general response. **500/concrete:** The number of supporters required for the proposal to be reviewed is 500, and the city government discloses a concrete action plan and timeline. **Interpretive implication:** Listing the four versions separately clarifies that respondents were assigned to one condition, rather than being asked to compare alternatives.
**Demographics and controls**	BG1. 거주지역: 수도권/비수도권 BG2. 성별: 남성/여성 BG3. 연령대: 18–29/30–44/45–59/60세 이상 BG4. 교육수준: 고졸/대학(재)/대학원 이상 BG5. 디지털 활용능력 (1 = 매우 낮음 … 7 = 매우 높음) BG6. 정치성향 (0 = 보수 … 10 = 진보)	BG1. Residential region: capital region/non-capital region BG2. Gender: male/female BG3. Age group: 18–29/30–44/45–59/60 or older BG4. Education level: high school graduate/university enrolled or graduate/graduate school or higher BG5. Digital ability (1 = very low … 7 = very high) BG6. Political orientation (0 = conservative … 10 = progressive) **Interpretive implication:** These variables support demographic adjustment and the digital-ability resource measure.
**Remaining documentation limits**	Not applicable; this row is a reporting note rather than original questionnaire wording.	Available materials do not report exact start and end field dates, recruitment panel or sampling frame, response/completion rates, compensation, public repository/access terms, formal IRB approval or exemption number, full consent-form wording, or detailed attention-check/exclusion wording. These omissions limit population generalization and prevent a complete audit of recruitment, consent, and data-quality procedures.

## Appendix B

### Robustness and Supplemental Diagnostics

This appendix consolidates the available robustness information within the manuscript, so the empirical claims are traceable without relying on separate supplemental files.

**Table A2:** Available Robustness Checks and Diagnostic Results.

Check	Available Result	Conclusion for Interpretation
Condition means	Scenario means are 4.43 (50/generic), 4.30 (50/concrete), 4.30 (500/generic), and 4.46 (500/concrete).	No visible condition separation is evident.
OLS full model	Baseline participation intention (b = 0.333, *p* < 0.001), outcome efficacy (b = 0.265, *p* = 0.001), and digital ability (b = 0.160, *p* = 0.001) are the strongest predictors; treatment terms are non-significant.	Measured belief/resource variables are associated with scenario willingness; causal claims remain limited to randomized treatments.
Multicollinearity	Largest construct correlations are r = 0.76; all VIF values are below 5, with a maximum of 3.92 for administrative responsiveness.	Multicollinearity is moderate but not fatal.
SEM fit	CFI = 0.954, TLI = 0.947, RMSEA = 0.047, SRMR = 0.066; chi-square is significant.	Approximate fit is acceptable; significant chi-square is expected with *N* = 500 and many indicators.
SEM path pattern	Latent participation intention and latent outcome efficacy are significantly associated with scenario willingness; experimental treatment terms remain non-significant.	The latent model reproduces the OLS interpretation while accounting for measurement error.
Ordered-logit robustness	Procedural treatments remain non-significant; baseline participation intention, outcome efficacy, and digital ability remain significant predictors.	The conclusion is not driven by treating the 7-point outcome as interval-scaled.
Outcome-efficacy robustness	Item-deletion and single-item specifications preserve the substantive association.	The outcome-efficacy result is not solely an artifact of the three-item composite.

**Table A3:** SEM Approximate-Fit Statistics Reported in the Manuscript.

Fit Statistic	Value	Interpretation
Comparative Fit Index (CFI)	0.954	Above the commonly used 0.95 benchmark for good approximate fit.
Tucker–Lewis Index (TLI)	0.947	Near the commonly used 0.95 benchmark.
Root Mean Square Error of Approximation (RMSEA)	0.047	Below 0.05, indicating close approximate fit.
Standardized Root Mean Square Residual (SRMR)	0.066	Below 0.08, indicating acceptable residual fit.
Chi-square test	Significant	Interpreted cautiously because chi-square is sensitive to sample size and model complexity.

## Appendix C

### Sample Transparency and Reporting Status

This appendix records which sample-transparency details are available in the study documentation. Items marked as not reported are not presented as missing placeholders in the article text, but they remain limits on what the study can claim about population representation and research governance.

**Table A4:** Sample Transparency and Reporting Status.

Reporting Field	Status and Effect on Manuscript Claims	Reporting Field
Sample size	**Status in available documentation:** Reported: *N* = 500 adults in South Korea. **Effect on manuscript claims:** Supports survey-based estimation within the analytic sample.	Sample size
Gender balance	**Status in available documentation:** Reported: 251 male (50.2%) and 249 female (49.8%). **Effect on manuscript claims:** Descriptive balance by gender.	Gender balance
Region balance	**Status in available documentation:** Reported: 250 capital-region respondents and 250 non-capital-region respondents. **Effect on manuscript claims:** Descriptive balance by broad region.	Region balance
Education profile	**Status in available documentation:** Reported: 384 respondents (76.8%) have college education or higher. **Effect on manuscript claims:** Population generalization is limited because education is high.	Education profile
Experimental-cell balance	**Status in available documentation:** Reported: four cells of 125 respondents each. **Effect on manuscript claims:** Supports clean internal comparison of randomized vignette conditions.	Experimental-cell balance
Weighting	**Status in available documentation:** Analyses are unweighted. **Effect on manuscript claims:** Population estimates are not claimed.	Weighting
Organizing institution and survey agency	**Status in available documentation:** Reported: Korea Local Administration Institute as organizing institution and Research Lab Co., Ltd. as survey agency. **Effect on manuscript claims:** Identifies the institutional/survey source, but does not identify the recruitment panel or sampling frame.	Organizing institution and survey agency
Survey instrument date and expected response time	**Status in available documentation:** Reported: survey instrument dated November 2025; expected response time approximately 12–15 min. **Effect on manuscript claims:** Provides month-level documentation and approximate burden, but not exact start and end field dates.	Survey instrument date and expected response time
Recruitment panel or sampling frame	**Status in available documentation:** Not reported in available documentation. **Effect on manuscript claims:** Panel representativeness cannot be assessed.	Recruitment panel or sampling frame
Exact field dates	**Status in available documentation:** Exact start and end dates are not reported in available documentation. **Effect on manuscript claims:** Temporal context cannot be fully assessed.	Exact field dates
Response/completion rate	**Status in available documentation:** Not reported in available documentation. **Effect on manuscript claims:** Nonresponse and completion bias cannot be assessed.	Response/completion rate
Compensation	**Status in available documentation:** Not reported in available documentation. **Effect on manuscript claims:** Incentive effects cannot be assessed.	Compensation
